# Standard care vs. TRIVEntricular pacing in Heart Failure (STRIVE HF): a prospective multicentre randomized controlled trial of triventricular pacing vs. conventional biventricular pacing in patients with heart failure and intermediate QRS left bundle branch block

**DOI:** 10.1093/europace/euab267

**Published:** 2021-11-22

**Authors:** Justin Gould, Simon Claridge, Thomas Jackson, Benjamin J Sieniewicz, Baldeep S Sidhu, Bradley Porter, Mark K Elliott, Vishal Mehta, Steven Niederer, Humra Chadwick, Ravi Kamdar, Shaumik Adhya, Nikhil Patel, Shoaib Hamid, Dominic Rogers, William Nicolson, Cheuk F Chan, Zachary Whinnett, Francis Murgatroyd, Pier D Lambiase, Christopher A Rinaldi

**Affiliations:** 1 Guy’s & St. Thomas’ Hospitals, Westminster Bridge Road, London, SE1 7EH, UK; 2 King’s College London, Westminster Bridge Road, London, SE1 7EH, UK; 3 Croydon University Hospital, 530, London Road, Croydon, CR7 7YE, UK; 4 Maritime Hospital, Windmill Road, Gillingham, Kent, ME7 5NY, UK; 5 Eastbourne District General Hospital, King's Drive, Eastbourne, East Sussex, BN21 2UD, UK; 6 Queen Elizabeth Hospital, Stadium Road, London, SE18 4QH, UK; 7 Northen General Hospital, Herries Road, Sheffield, South Yorkshire, S5 7AU, UK; 8 Glenfield Hospital, Groby Road, Leicester, Leicestershire, LE3 9QP, UK; 9 East Surrey Hospital, Canada Avenue, Redhill, RH1 5RH, UK; 10 Hammersmith Hospital, Du Cane Road, London W12 0HS, UK; 11 King’s College Hospital, Denmark Hill, London, SE5 9RS, UK; 12 Barts Heart Centre, St Bartholomew's Hospital, West Smithfield, City of London, EC1A 7BE, UK

**Keywords:** Cardiac resynchronization therapy, Multi-site pacing, Multi-lead left ventricular pacing, Triventricular pacing, Improving cardiac resynchronization therapy response

## Abstract

**Aims:**

To determine whether triventricular (TriV) pacing is feasible and improves CRT response compared to conventional biventricular (BiV) pacing in patients with left bundle branch block (LBBB) and intermediate QRS prolongation (120–150 ms).

**Methods and results:**

Between October 2015 and November 2019, 99 patients were recruited from 11 UK centres. Ninety-five patients were randomized 1:1 to receive TriV or BiV pacing systems. The primary endpoint was feasibility of TriV pacing. Secondary endpoints assessed symptomatic and remodelling response to CRT. Baseline characteristics were balanced between groups. In the TriV group, 43/46 (93.5%) patients underwent successful implantation vs. 47/49 (95.9%) in the BiV group. Feasibility of maintaining CRT at 6 months was similar in the TriV vs. BiV group (90.0% vs. 97.7%, *P *=* *0.191). All-cause mortality was similar between TriV vs. BiV groups (4.3% vs. 8.2%, *P *=* *0.678). There were no significant differences in echocardiographic LV volumes or clinical composite scores from baseline to 6-month follow-up between groups.

**Conclusion:**

Implantation of two LV leads to deliver and maintain TriV pacing at 6 months is feasible without significant complications in the majority of patients. There was no evidence that TriV pacing improves CRT response or provides additional clinical benefit to patients with LBBB and intermediate QRS prolongation and cannot be recommended in this patient group.

**Clinical trial registration number:**

Clinicaltrials.gov: NCT02529410.

What’s new?

- Standard care vs. TRIVEntricular pacing in Heart Failure (STRIVE HF) is the first randomized multicentre trial designed to evaluate the feasibility, safety, and clinical value in improving cardiac resynchronization therapy (CRT) response of Triventricular (TriV) compared to conventional biventricular pacing in patients undergoing CRT-defibrillator implantation with Class IB indications for CRT [left bundle branch block (LBBB) QRS 120–150 ms].
- STRIVE HF is the largest randomized multicentre study of dual left ventricular (LV) lead pacing in CRT naïve patients.
- Implantation of two transvenous LV leads via the coronary sinus is feasible and safe in the short term.
- Delivery of TriV pacing was feasible at 6-month follow-up in the majority of patients.
- There was no evidence that TriV pacing improved CRT response or provided any clinical benefit to patients with LBBB and intermediate QRS prolongation.
- Procedure times were longer and battery longevity was reduced in the TriV group. The current study therefore does not support the practice of multi-lead LV pacing in this patient group.

## Introduction

Cardiac resynchronization therapy (CRT) improves symptoms and prognosis in selected patients with dysynchronous heart failure.^1–5^ However, a significant proportion of patients (30–50%) do not derive clinical benefit or show evidence of reverse remodelling.^3^^,^^6^ Poor patient selection, suboptimal left ventricular (LV) lead positioning, and insufficient delivery of CRT are important causes of CRT non-response.^7–9^ Meta-analyses from randomized trials demonstrate CRT is most effective in patients with QRS duration ≥150 ms and that CRT may not reduce events in patients with QRS <150 ms.^10^ This is reflected within guidelines where the strongest evidence for benefit is in patients with left bundle branch block (LBBB) with QRS >150 ms (Class IA recommendation) and a lower level of recommendation (Class IB) for patients with LBBB with QRS 120–150 ms.^11^ Since these intermediate QRS LBBB patients represent at least 20% of heart failure cases, it is important to optimize therapy in this group.^12^ A multi-lead LV pacing strategy (multi-site pacing using two LV leads) may improve CRT response by increasing the probability of capturing more LV myocardium potentially providing faster and more physiological LV activation.^13^ Multi-lead LV pacing has the potential advantage over multi-point pacing using a quadripolar lead in that it allows a theoretical larger separation of two LV electrodes and may allow simultaneous recruitment of a larger volume of viable LV myocardium compared to single- or multi-point LV pacing.^14^ Multi-lead LV pacing may capture the myocardium around areas of scar more effectively resulting in an improvement in CRT response.^14–18^ In contrast, pathophysiological work has demonstrated a negligible benefit with increasing the number of LV pacing sites when an adequate response is achieved with biventricular (BiV) pacing most likely because the lateral placement of the lead to a site of latest activation maximizes recruitment.^19^^,^^20^ However, patients with a lesser degree of QRS prolongation (QRS 120–150 ms) may potentially benefit from multi-lead LV pacing as there may be more discrete single sites to target. Since these patients are known to derive less benefit from CRT, alternative strategies need to be rigorously tested.

The STRIVE HF (Standard care vs. TRIVEntricular pacing in Heart Failure) trial was designed to examine whether triventricular (TriV) pacing [two LV leads, one right ventricular (RV lead)] in patients with LBBB with a moderately prolonged QRS duration of 120–150 ms was feasible and superior in terms of the proportion of patients responding to CRT compared to standard BiV pacing.

## Methods

Between October 2015 and November 2019, 99 patients were recruited from 11 UK centres. All participants provided written consent. The study protocol was approved by the South East Coast Research Ethical Committee (15/LO/0183) and conducted in accordance with the Declaration of Helsinki. An outline of the study including prespecified endpoints is available on Clinicaltrials.gov (Identifier: NCT02529410).

### Recruitment and follow-up

Consecutive patients undergoing CRT-defibrillator (CRT-D) implantation were screened for eligibility at each centre. Patients who had Class IB indication for CRT (LBBB QRS 120–150 ms) as per European Society of Cardiology (ESC) guidelines 2013^11^ were eligible for enrolment. Patients of any gender and ≥18 years old could participate providing they could comply with all study requirements and give consent. Patients who were pregnant, lactating, or planning pregnancy during the study were ineligible.

All study participants were on optimal heart failure/antiarrhythmic pharmacotherapy prior to device implantation (Supplementary material online, *Table SA*). Eligible patients underwent the following assessments at baseline and 6-month follow-up visits: New York Heart Association (NYHA) functional class assessment; physical examination; 12-lead resting electrocardiogram (ECG); two-dimensional (2D) transthoracic echocardiogram [including Simpson’s biplane assessment for LV end-diastolic/end-systolic volumes (LVEDV, LVESV) and left ventricular ejection fraction (LVEF)]; Minnesota living with heart failure questionnaire score (MLWHFQ); 6-min walk test (6MWT); and N-terminal pro-B-type natriuretic peptide (NTpro-BNP). All patients underwent device interrogation at 6-month follow-up.

### Randomization

**Figure 1:**
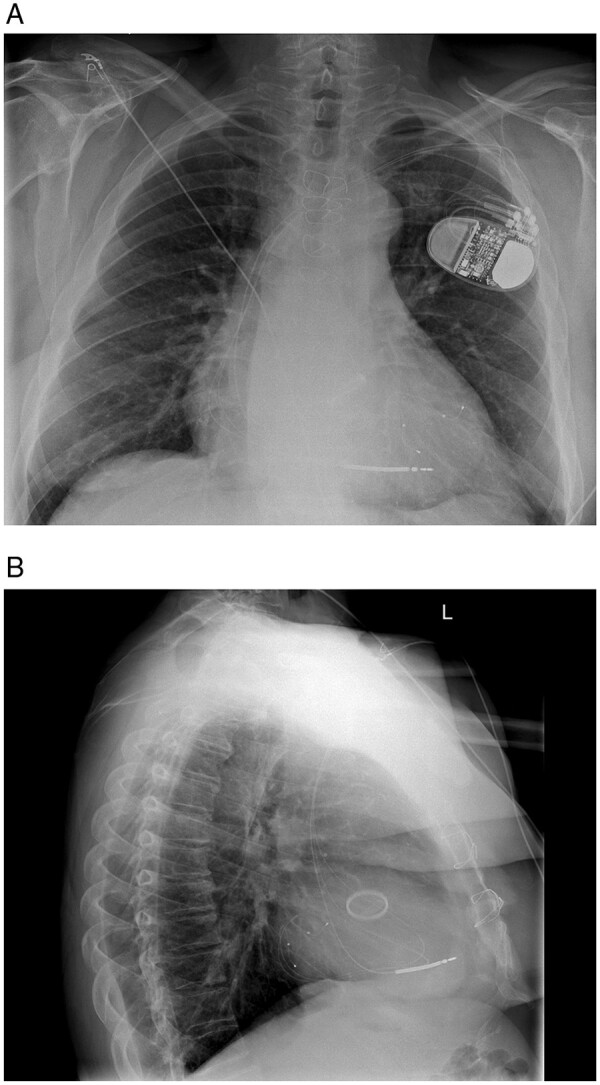
Representative posterior–anterior (*A*) and lateral (*B*) chest radiographs 1-day post-implantation showing a triventricular cardiac resynchronization therapy-defibrillator. Left ventricular leads are located in posterolateral and lateral coronary veins.

Enrolled patients were randomly assigned using a computerized minimization method (1:1 ratio) to receive either a TriV CRT-D [one RV shock lead, two LV leads with maximal possible lead separation ± right atrial (RA) lead (*Figure 1*)] or a conventional BiV CRT-D (one RV shock lead, one LV lead ± RA lead) and were stratified according to clinical centre; ischaemic (ICM) or non-ischaemic cardiomyopathy (NICM); sinus rhythm; or permanent atrial fibrillation (AF).

### Two-dimensional transthoracic echocardiography

Left ventricular end-diastolic volume and LVESV were derived by averaging volumes from two- and four-chamber windows using modified Simpson’s biplane and LVEF calculated.

### Implantation

One RV lead was deployed to the septum or apex according to operator preference/optimal lead parameters in both groups. An RA lead was deployed to the RA appendage for patients predominantly in sinus rhythm. For patients randomized to TriV implantation, two bipolar LV leads were implanted transvenously via the coronary sinus (CS). Operators performed two LV lead implantation using two guide catheters via separate venous access or by using the Worley™ Advance Coronary Sinus Guide and LV lead delivery system (Merit Medical, South Jordan, UT, USA) allowing delivery of two LV leads via a single guide sheath. Operators were instructed to aim for maximal LV lead separation as permitted by optimal lead parameters and the absence of phrenic nerve stimulation (PNS). The first LV lead (LV_1_) was targeted to a posterolateral or lateral vein and the second LV lead (LV_2_) as far as anatomically possible from LV_1_, in an anterior, anterolateral, or middle cardiac vein as governed by individual coronary venous anatomy. The two bipolar LV leads were connected to a TriV device via two dedicated IS-1 ports with an internal parallel Y-port (Paradym TriV CRT-D, ICV1231, MicroPort CRM, Clarmart, France). A single LV output was programmed for all patients (acceptable thresholds were required for both LV pacing leads given individual LV outputs were not programmable). Patients in the BiV group received a quadripolar LV lead (Quartet, St. Jude Medical, St. Paul, MN, USA); LV vectors were selected based on optimal LV thresholds without PNS. Following implantation, both groups were programmed with atrioventricular (AV) delays of 100 ms and simultaneous RV–LV pacing.

### Endpoints

The primary endpoint was feasibility of achieving and maintaining TriV pacing at 6 months, calculated as the percentage of surviving patients still TriV pacing at 6 months based on device interrogation.

### Secondary endpoints

1. Proportionate effect of TriV vs. BiV pacing on reverse remodelling (comparison of percentage reduction in LVESV).
2. Proportion of patients who reverse remodelled (defined as a reduction in LVESV ≥15% derived from 2D echocardiogram).
3. Proportionate effect of TriV vs. BiV pacing on reverse remodelling (comparison of percentage reduction in LVESV) in patients with prespecified subgroups of AF and heart failure aetiology.
4. Proportion of patients who reverse remodelled (defined as a reduction in LVESV ≥15%) in patients with prespecified subgroups of AF and heart failure aetiology.
5. Mean change and percentage change in NTpro-BNP in patients with TriV vs. BiV devices.
6. Comparison of TriV and BiV pacing on scores in the MLWHFQ.
7. Comparison of effect of TriV and BiV pacing on change in 6MWT (m).
8. Comparison of implantable cardioverter-defibrillator (ICD) shock therapy in TriV vs. BiV arm.

Other prespecified secondary outcome measures included the Packer clinical composite score,^21^ time to first heart failure hospitalization, rates of adverse events, and mortality during the study period. All adverse events were reported and adjudicated by the chief investigator and sponsor (Guy’s & St. Thomas’ Hospitals) who reviewed the event type, severity, and relatedness to an additional LV lead implant.

### Statistics

Data analysis was performed according to intention-to-treat principles. Discrete data are presented as *n* values (percentages); continuous data as mean ± 1 SD and/or median (interquartile range). Discrete variables were compared using the Fisher’s exact test. Continuous data were assessed for normality with the Shapiro–Wilk test where *P*-value ≥0.05 was considered normally distributed data. Normally distributed data were compared with an independent samples *t*-test. Non-normally distributed data were compared using the Wilcoxon signed-rank test. All statistical tests were two-sided and *P*-value <0.05 was considered statistically significant. Statistical analysis was performed using Statistical Package for Social Sciences, Macintosh, V24.0.0.1 (2017), Armonk, NY, USA: IBM, and GraphPad Prism v9.0.0, GraphPad Software, San Diego, CA, USA.

## Results

**Figure 2:**
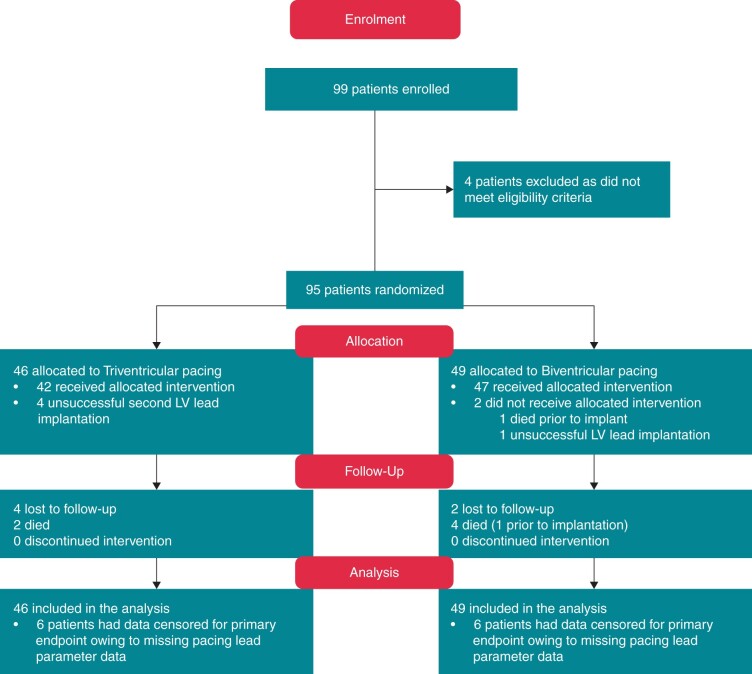
Standard care vs. TRIVEntricular pacing in Heart Failure (STRIVE HF) CONSORT flow diagram. CONSORT diagram adapted from Schulz *et al*.^22^ LV, left ventricular.

Ninety-nine out of a target 100 patients were enrolled. Four patients were excluded prior to implant due to not meeting eligibility criteria (*Figure 2*, CONSORTdiagram^22^). Ninety-five patients underwent randomization (TriV group *n* = 46; BiV control group *n* = 49). Baseline characteristics and pharmacological therapy were balanced between both groups (*Table 1* and Supplementary material online, *Table SA*). In the TriV group 42/46 (91.3%), patients were successfully implanted with a TriV system. In four patients, the second LV lead could not be sited and they received a single LV lead BiV system. In the BiV group, 47/49 (95.9%) patients were successfully implanted with a BiV system; one patient died following randomization but prior to implantation and another failed transvenous LV lead implantation and received a dual-chamber ICD.

**Table 1:** Baseline characteristics

Characteristic	Triventricular group	Biventricular group	All patients	*P-*value
Age (years)	69.0 ± 9.9 (*n* = 46)	67.9 ± 9.8 (*n* = 49)	68.4 ± 9.8 (*n* = 95)	0.596
Male	36/46 (78.3)	36/49 (73.5)	72/95 (75.8)	0.638
Ischaemic cardiomyopathy	25/46 (54.3)	30/49 (61.2)	55/95 (57.9)	0.538
Previous coronary artery bypass surgery	5/46 (10.9)	9/49 (18.4)	14/95 (14.7)	0.390
Previous valve surgery	4/46 (8.7)	4/49 (8.2)	8/95 (8.4)	1.000
Hypercholesterolemia	5/46 (10.9)	12/49 (24.5)	17/95 (17.9)	0.110
Current tobacco smoking	6/46 (13.0)	3/49 (6.1)	9/95 (9.5)	0.307
Previous tobacco smoking	6/46 (13.0)	13/49 (26.5)	19/95 (20.0)	0.127
Diabetes mellitus	17/46 (37.0)	22/49 (44.9)	39/95 (41.1)	0.604
Hypertension	15/46 (32.6)	20/49 (40.8)	35/95 (36.8)	0.524
Atrial fibrillation	11/46 (23.9)	12/49 (24.5)	23/95 (24.2)	1.000
QRS (ms)	135.7 ± 9.2 (*n* = 46)	137.2 ± 8.1 (*n* = 49)	136.5 ± 8.6 (*n* = 95)	0.474
LV ejection fraction (%)	26.1 ± 6.6 (*n* = 46)	27.3 ± 6.8 (*n* = 49)	26.7 ± 6.8 (*n* = 95)	0.408
LV end-diastolic volume (mL)	195.3 ± 88.4 (*n* = 44)	184.1 ± 63.5 (*n* = 48)	189.5 ± 76.2 (*n* = 92)	0.988
LV end-systolic volume (mL)	149.2 ± 76.1 (*n* = 44)	137.0 ± 58.8 (*n* = 48)	142.8 ± 67.6 (*n* = 92)	0.684
Impaired right ventricular systolic function	14/42 (33.3)	13/43 (30.2)	27/85 (31.8)	0.818
Systolic blood pressure (mmHg)	118.8 ± 14.4 (*n* = 43)	126.0 ± 18.6 (*n* = 42)	122.4 ± 16.9 (*n* = 85)	0.050

Values are presented as mean ± SD (*n* = number available for analysis) or as *n*/number available for analysis (%).

LV, left ventricular; SD, standard deviation.

**Figure 3:**
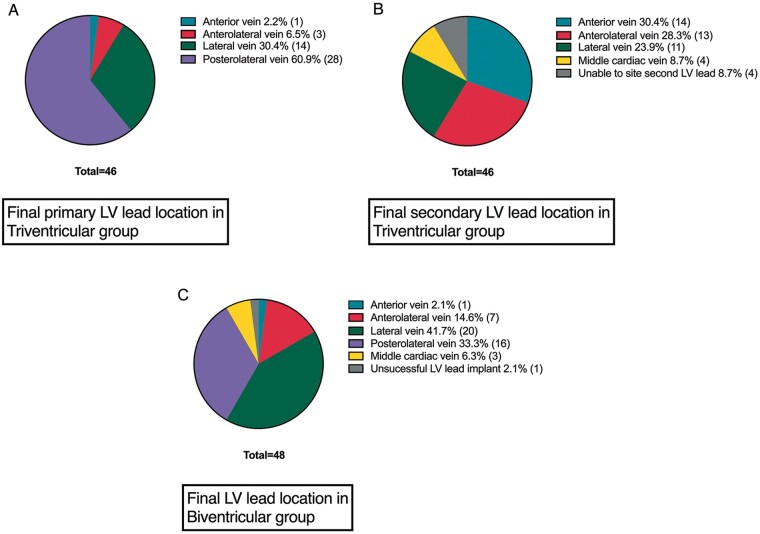
Pie charts showing the distribution of final primary (*A*) and secondary (*B*) left ventricular lead locations in the TriV group and in the biventricular group (*C*) determined by coronary venous anatomy. LV, left ventricular.

**Figure 4:**
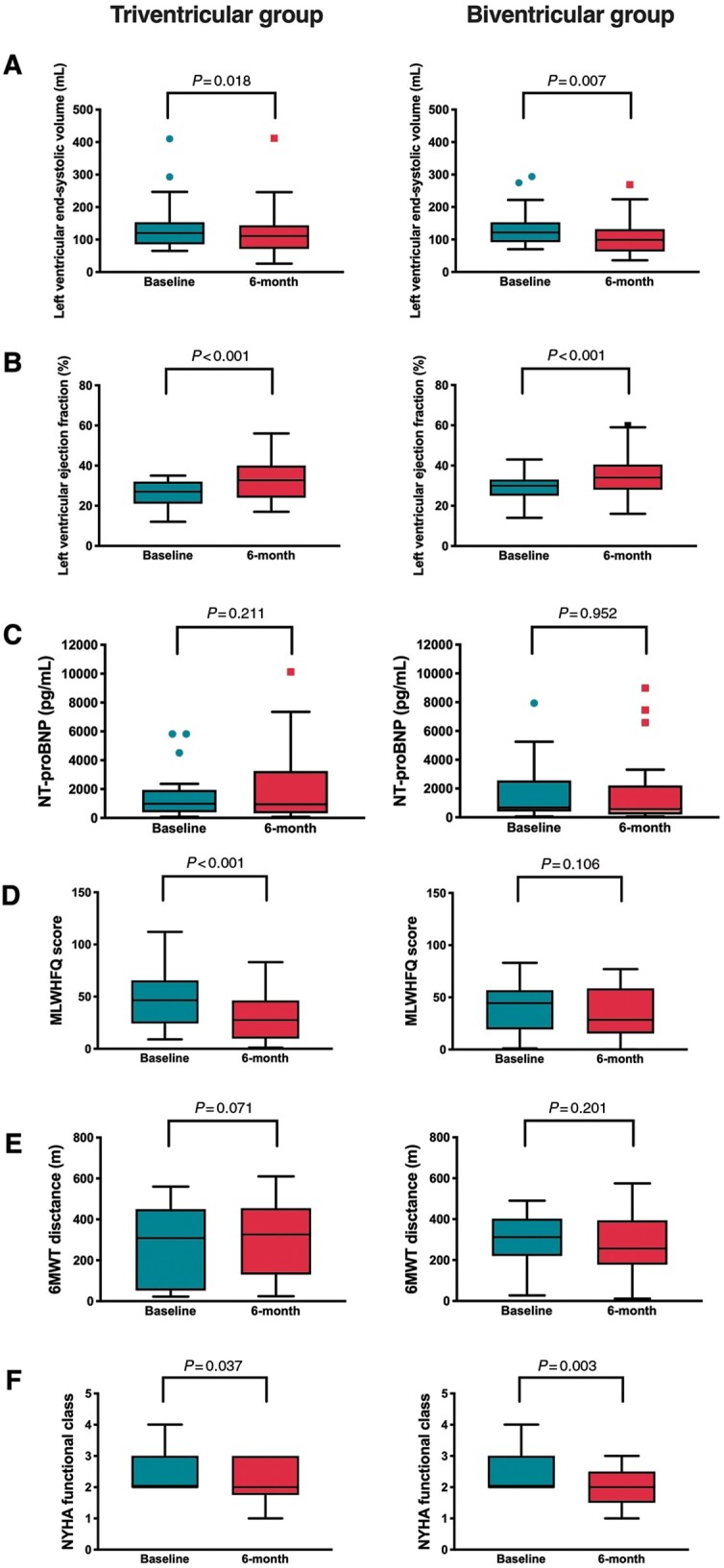
Box and whisker plots comparing echocardiographic and clinical measures at baseline and 6-month follow-up for both groups. A) Left ventricular end-systolic volume, B) Left ventricular ejection fraction, C) NT-proBNP, D) MLWHFQ score, E) 6MWT distance, F) NYHA functional class. LVEF, left ventricular ejection fraction; LVESV, left ventricular end-systolic volume; MLWHFQ, Minnesota living with heart failure questionnaire; NT-proBNP, N-terminal pro-B-type natriuretic peptide; NYHA, New York Heart Association; 6MWT, 6-min walk test.

*Figure 3* shows the distribution of final primary and secondary LV lead locations in the TriV group and in the BiV group determined by coronary venous anatomy. Procedure duration was significantly longer in the TriV group (192.6 ± 107.6 vs. 133.9 ± 50.9 min, *P *<* *0.001) as was the mean duration from CS intubation to final LV lead placement (72.2 ± 40.1 vs. 49.2 ± 36.6, *P *=* *0.002). Mean fluoroscopy times were significantly longer in the TriV compared to BiV group (36.9 ± 19.1 vs. 26.5 ± 15.8 min, *P *=* *0.004). Radiation dose area products were non-significantly higher in the TriV group (3169 ± 3401 vs. 2425 ± 2252 cGycm^2^, *P *=* *0.545). Mean contrast volume was non-significantly higher in the TriV vs. BiV groups (88.8 ± 56.4 vs. 67.7 ± 47.6 mL, *P *=* *0.075). Mean LV pacing thresholds at implant were significantly higher in the TriV group (1.3 ± 0.5 vs. 1.0 ± 0.5 V, *P *=* *0.004) with similar LV lead pulse widths between the TriV and BiV groups (0.5 ± 0.2 vs. 0.5 ± 0.1 ms, *P *=* *0.903). Left ventricular lead impendences were significantly lower in the TriV group (735 ± 286 vs. 864 ± 315 Ω, *P *=* *0.044). There were two reported lead displacements: one RV lead displacement in the TriV group and one LV lead displacement in the BiV group, both of which were re-sited within the study period. There was a limited CS dissection in one patient in the TriV group with no sequela (the patient received and maintained TriV pacing at 6 months). Within both the TriV and BiV groups, patients displayed a significant reduction in LVESV and increase in LVEF at 6 months compared to baseline, indicating that patients within both groups did reverse remodel and respond to CRT (*Figure 4*).

There were no significant differences in all-cause mortality, heart failure hospitalization, other cardiovascular hospitalization, or a composite of all three (*Table 2*). There were six deaths during the study. Two in the TriV group due to end-stage heart failure prior to their 6-month review. One patient randomized to the BiV group died prior to CRT-D implantation and two further patients in the BiV group died due to bronchopneumonia remote from their CRT implantation. One patient in the BiV group died from sepsis and multi-organ failure a week following CRT-D implantation; in view of the temporal relation this was felt to be procedure related.

**Table 2:** Feasibility and safety of TriV and BiV pacing

Variable	TriV group	BiV group	All patients	*P-*value
Feasibility of maintaining BiV/TriV pacing^a^	36/40 (90.0)	42/43 (97.7)	78/83 (94.0)	0.191
All-cause mortality	2/46 (4.3)	4/49 (8.2)	6/95 (6.3)	0.678
HF hospitalization	2/40 (5.0)	1/42 (2.4)	3/82 (3.7)	0.611
Other CV hospitalization	4/40 (10.0)	2/42 (4.8)	6/82 (7.3)	0.427
Composite all-cause mortality/HF and other CV hospitalization	8/46 (17.4)	7/49 (14.3)	15/95 (15.8)	0.781
Appropriate ICD shock therapy (%)	1/40 (2.5)	1/42 (2.4)	2/82 (2.4)	1.000

Values are presented as *n*/number available for analysis (%). Feasibility of achieving and maintaining BiV/TriV pacing at 6 months calculated as the percentage of surviving patients followed up at 6 months and still TriV or BiV pacing at 6 months based on their 6-month pacing check.

BiV, biventricular; CV, cardiovascular; HF, heart failure; ICD, implantable cardioverter-defibrillator; TriV, triventricular.

a No feasibility data was available for four patients in the TriV group and two patients in the BiV group due to loss to follow-up and therefore these patients were excluded from feasibility analysis (two patients in each group were lost to follow-up due to COVID-19 restrictions preventing a full 6-month research follow-up appointment). A further six patients died prior to their 6-month follow-up (1 prior to implant) and were excluded for this feasibility of maintaining TriV/BiV pacing analysis only.

### Primary endpoint

Feasibility of achieving and maintaining CRT at 6 months was similar between TriV and BiV groups (90.0%, *n* = 36/40 vs. 97.7%, *n* = 42/43, *P *=* *0.191) (*Table 2*).

### Secondary endpoints

1. There was no significant difference in absolute or percentage change of LVESV from baseline to 6-month follow-up between TriV and BiV groups (Table 3).
2. There was no significant difference in the number of patients that reverse remodelled (i.e. the number of volumetric responders) between TriV and BiV groups (32.6% vs. 42.9%, P = 0.398) (Supplementary material online, Table SB).
3. There was no significant difference in absolute or percentage change of LVESV from baseline to 6-month follow-up between TriV and BiV groups in patients with sinus rhythm, AF, ICM, or NICM (Supplementary material online, Table SC).
4. There was no significant difference in the number of patients that reverse remodelled between TriV and BiV groups in patients with sinus rhythm, AF, ICM, or NICM (Supplementary material online, Table SB).
5. There was no significant difference in absolute or percentage change of NTpro-BNP from baseline to 6-month follow-up between TriV and BiV groups (Table 3).
6. There was no significant difference in absolute or percentage change in MLWHFQ scores from baseline to 6-month follow-up between TriV and BiV groups (Table 3).
7. There was no significant difference in absolute or percentage change of 6MWT distance from baseline to 6-month follow-up between TriV and BiV groups (Table 3).
8. The mean number of ICD shock therapies was similar between TriV vs. BiV groups (2.4%, n = 1 vs. 2.5%, n = 1, P = 1.000) (Table 2).

**Table 3:** Echocardiographic and clinical measures

Variable	Triventricular group	Biventricular group	*P-*value
LV end-diastolic volume (mL)	*n* = 38	*n* = 39	
Baseline	183.6 ± 88.2	181.4 ± 58.6	
	169.0 (123.3–192.0)	173.0 (137.0–212.0)	
Follow-up	171.9 ± 81.0	158.5 ± 62.4	
	173.6 (119.5–202.5)	144.0 (104.0–193.0)	
Absolute change (mL)	−11.7 ± 52.3	−22.9 ± 57.0	0.105
	3.0 (−26.5 to 22.4)	−20.1 (−61.0 to 12.0)	
Percentage change (%)	−4.6 ± 21.9	−10.2 ± 29.7	0.350
	1.5 (−19.8 to 10.0)	−15.3 (−33.6 to 8.1)	
LV end-systolic volume (mL)	*n* = 37	*n* = 39	
Baseline	134.3 ± 68.7	131.6 ± 51.7	
	120.0 (85.5–153.5)	122.0 (92.0–153.0)	
Follow-up	118.6 ± 69.1	106.7 ± 53.1	
	111.0 (71.5–144.0)	98.9 (63.0–132.0)	
Absolute change (mL)	−15.8 ± 38.9	−24.9 ± 56.5	0.356
	−12.0 (−33.5 to 12.2)	−21.4 (−52.0 to 4.5)	
Percentage change (%)	−11.8 ± 25.9	−14.8 ± 38.5	0.691
	−9.8 (−29.5 to 9.4)	−18.1 (−41.6 to 5.2)	
LV ejection fraction (%)	*n* = 39	*n* = 41	
Baseline	26.1 ± 6.8	28.6 ± 6.0	
	27.0 (21.0–32.0)	30.0 (25.0–33.0)	
Follow-up	32.5 ± 10.2	36.0 ± 10.2	
	32.7 (24.0–40.0)	34.0 (28.0–40.5)	
Absolute change (%)	6.4 ± 9.3	7.3 ± 10.2	0.676
	7.0 (−2.0 to 12.0)	5.0 (0.5–16.5)	
NT-proBNP (pg/mL)	*n* = 25	*n* = 27	
Baseline	1503.3 ± 1620.4	1638.8 ± 2004.1	
	980.0 (391.5–1947.0)	686.0 (402.0–2568)	
Follow-up	2115.2 ± 2678.9	1660.8 ± 2395.7	
	942.0 (326.0–3259.5)	561 (196.0–2214.0)	
Absolute change	612.0 ± 2380.0	22.1 ± 1887.9	0.128
	105.0 (−370.0 to 1107.0)	−30.0 (−682.0 to 106.0)	
Percentage change (%)	66.9 ± 146.9	85.4 ± 460.8	0.092
	29.3 (−25.0 to 93.2)	−6.0 (−52.2 to 20.4)	
MLWHFQ (score)	*n* = 34	*n* = 36	
Baseline	46.7 ± 25.4	40.1 ± 22.7	
	46.5 (24.3–65.5)	44.5 (19.3–56.8)	
Follow-up	30.7 ± 23.2	34.5 ± 23.3	
	27.5 (9.8–46.3)	28.5 (15.3–58.5)	
Absolute change	−16.0 ± 24.4	−5.5 ± 20.0	0.054
	−15.0 (−31.5 to −2.5)	−8.0 (−17.8 to 5.0)	
Percentage change (%)	−21.9 ± 65.7	6.7 ± 98.5	0.177
	−35.2 (−68.0 to −3.2)	−21.9 (−52.0 to 22.3)	
Six-minute walk test (m)	*n* = 27	*n* = 29	
Baseline	274.0 ± 191.0	305.2 ± 139.5	
	308.0 (52.0–450.0)	312.0 (220.1–402.5)	
Follow-up	305.2 ± 181.3	275.2 ± 154.5	
	326.1 (130.0–455.0)	256.0 (177.5–394.5)	
Absolute change (m)	31.2 ± 86.0	−29.9 ± 122.9	0.051
	10.9 (−26.0 to 50.0)	−8.5 (−61.1 to 22.5)	
Percentage change (%)	67.4 ± 219.7	−5.4 ± 42.2	0.066
	4.0 (−11.1 to 17.9)	−2.7 (−20.8 to 10.6)	
New York Heart Association class	*n* = 38	*n* = 41	
Baseline	2.4 ± 0.5	2.3 ± 0.5	
	2.0 (2.0–3.0)	2.0 (2.0–3.0)	
Follow-up	2.1 ± 0.8	2.0 ± 0.7	
	2.0 (1.8–3.0)	2.0 (1.5–2.5)	
Absolute change	−0.2 ± 0.7	−0.3 ± 0.7	0.762
	0.0 (−1.0 to 0.0)	0.0 (−1.0 to 0.0)	
Percentage change (%)	−9.4 ± 31.5	−13.4 ± 27.9	0.715
	0.0 (−37.5 to 0.0)	0.0 (−50.0 to 0.0)	

All values are presented as mean ± SD and median (IQR) with (*n* = number available for analysis). Absolute and percentage change values are the difference between values obtained from baseline pre-assessment and 6-month follow-up measurements.

IQR, interquartile range; LV, left ventricular; MLWHFQ, Minnesota living with heart failure questionnaire; NT-proBNP, N-terminal pro-B-type natriuretic peptide; SD, standard deviation.

In terms of the Packer clinical composite score,^21^ there was no significant difference in the number of patients that improved (35.0% vs. 31.8%, *P *=* *0.819), remained unchanged (42.5% vs. 54.5%, *P *=* *0.285) or worsened (22.5% vs. 13.6%, *P *=* *0.394) between TriV and BiV groups. Furthermore, there was no significant difference in absolute change or percentage change of LVEDV and LVEF values from baseline to 6-month follow-up between TriV and BiV groups (*Table 3*). Battery longevity (defined as the mean elective replacement index) was significantly lower in the TriV group (5.5 ± 2.3 vs. 8.6 ± 2.7 years, *P* = <0.001).

## Discussion

STRIVE HF is the first randomized multicentre trial designed to evaluate the feasibility, safety, and clinical value in improving CRT response of TriV compared to conventional BiV pacing in patients undergoing CRT-D implantation with Class IB indications for CRT (LBBB QRS 120–150 ms).^11^ STRIVE HF is the largest randomized multicentre study of dual LV lead pacing in CRT naïve patients.

Implanting two LV leads and maintaining TriV pacing at 6 months was feasible in 90.0% of patients and was similar compared to the feasibility of maintaining BiV pacing in 97.7% of the control group. Two LV leads were successfully implanted in 43/46 (93.5%) patients in the TriV group. In the three patients where addition of a second LV lead was not technically possible, these patients received a BiV pacing system with a single LV lead instead. In one patient, this was due to high LV thresholds and PNS in the available coronary veins for a second LV lead; in the other two patients, the attempt at adding a second LV lead was abandoned due to failed CS cannulation for the second lead. The study protocol advised maximal LV lead separation, however, placing a single LV lead in the coronary venous system can be technically challenging with complicated coronary venous anatomy, poor lead stability, suboptimal LV pacing thresholds, or presence of PNS. Despite this, placing two LV leads in the coronary venous circulation was achievable with delivery of TriV pacing in the majority of patients (90%).

### Safety and practicality of triventricular pacing 

The short- to medium-term safety profile of TriV pacing was acceptable with no recorded procedure-related deaths or procedure-related major complications in the TriV group. There were no reported device-related infections during the 6-month study period. The use of an internal Y-connector as opposed to an external Y-connector made implantation of the TriV system more straightforward for operators. TriV pacing resulted in longer procedure durations (driven by longer time spent deploying two LV leads in the coronary venous system) and longer mean fluoroscopy times. Threshold rises were observed in 18/40 (45.0%) patients in the TriV group at 6 months. TriV pacing was ‘deactivated’ in six patients after the 6-month study period by lowering LV lead outputs below the highest LV threshold to improve battery longevity, accepting some remaining battery drain in the ‘deactivated’ lead and therefore inferior to BiV pacing with optimal single LV lead thresholds.

**Figure 5:**
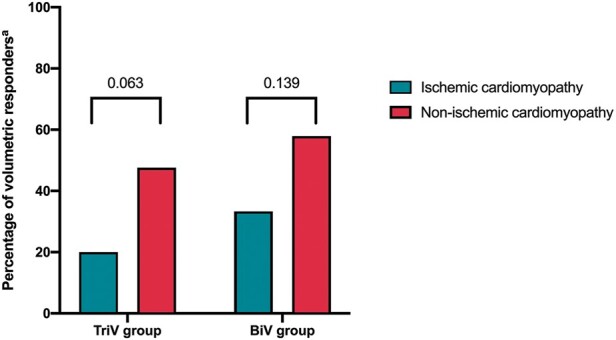
Cardiac resynchronization therapy volumetric response outcomes by heart failure aetiology within triventricular and biventricular groups. ^a^Volumetric response defined as ≥15% reduction in left ventricular end-systolic volume on two-dimensional echocardiography. BiV, biventricular; TriV, triventricular.

All primary and secondary endpoints were similar between TriV and BiV groups. There was no evidence of superior volumetric remodelling benefits in the TriV compared to BiV group. Volumetric response rates were non-significantly lower in the TriV (32.6%) vs. BiV group (42.9%) and together with a significantly shorter battery longevity (due to higher mean LV pacing thresholds), there was no evidence to support the use of TriV pacing in patients with a Class IB indication for CRT (LBBB QRS 120–150 ms).^11^ This remained the case in prespecified subgroups of patients with sinus rhythm, permanent AF, ICM, and NICM. Patients with ICM who could be hypothesized to have an incremental benefit with multi-lead LV pacing had an expectedly low volumetric remodelling response rate that was not improved with TriV pacing (*Figure 5*).

### Comparison with prior studies

Initial studies of multi-site pacing were for the most part undertaken in single centres in patients with mean QRS durations >150 ms and offered positive results compared to the present trial which only included patients with LBBB and intermediate QRS durations 120–150 ms. Lenarczyk *et al*.^16^ demonstrated the feasibility of TriV pacing in 22/26 patients (baseline mean QRS 169 ± 18 ms) with a >90% response rate at 3 months. In 34 patients with AF and a pre-existing indication for bradycardia pacing, Leclercq *et al*.^15^ compared TriV pacing with BiV pacing using two LV leads and one RV lead. The primary endpoint measure of ventricular resynchronization was unchanged; however, there were some improvements in remodelling secondary endpoints.^15^ Rogers *et al*.^17^ in a single-centre crossover study of 43 patients demonstrated a significant improvement in 6MWT, MLWHFQ scores, peak VO2, and LV ejection fraction at 6 months when comparing conventional BiV stimulation with TriV stimulation. This study had two TriV groups: Group A had two LV and one RV lead (baseline mean QRS 143 + 26 ms) and Group B had two RV and one LV lead (baseline mean QRS 134 + 39 ms). Notably, the improvement in echocardiographic parameters was powered by Group A rather than Group B which is in contrast to the findings in the present study. Ginks *et al*.^14^ reported multi-lead LV pacing increased the acute haemodynamic response rate to CRT in 16% of patients vs. single-site pacing but was only beneficial in patients with posterolateral scar identified on cardiac magnetic resonance imaging (study cohort baseline mean QRS 157 + 27 ms).

More recently, the V^3^ trial randomized 84 CRT non-responders according to their clinical composite scores to continued conventional BiV pacing (baseline mean paced QRS 155 ± 42 ms) or an upgrade to multi-lead LV pacing (baseline mean paced QRS 165 ± 31 ms).^23^ The V^3^ trial reported that TriV pacing was feasible with high implant success rates although addition of a second LV lead did not result in any significant clinical benefit or volumetric response in keeping with the current study.^23^ In the V^3^ trial, TriV pacing was associated with a significantly higher perioperative complication rate (20.4%).^23^ The V^3^ trial had a 2-year follow-up period compared to the present study (6 months) and recruited a sicker cohort of CRT non-responders involving the addition of a second LV lead which likely explains the higher number of complications. STRIVE HF also differs to the aforementioned studies as it is the first to use a dedicated generator capable of delivering multi-lead CRT without the need for an external Y-connector.

### Future directions

Acknowledging that patients with intermediate LBBB are poorer responders to CRT, other strategies need further exploration. For example, the emergence of His bundle and left bundle pacing may be alternatives, however, the overriding issue is the determination of the mechanism accounting for the conduction abnormalities then tailoring therapy accordingly. It is possible block simply exists at a specific level in the conduction system or at the Purkinje-myocardial interface or as a result of intramyocardial disease with diffuse or fixed fibrosis limiting activation wavefront progression.^24^ Each of these mechanisms will require different pacing solutions and the emergence of endocardial systems may overcome a number of these limitations enabling more rapid Purkinje-driven myocardial tissue recruitment. This trial failed to identify an overall benefit of TriV pacing in this population, however, subgroups of responders may exist. The challenge is to prospectively identify them utilizing ECG morphology, ECG imaging,^25^ cardiac imaging, or other strategies since this group represents 20% of all patients with heart failure.^12^

### Limitations

Patients and investigators were unblinded during follow-up due to intricacies assessing two LV lead thresholds in TriV devices which may have introduced bias. Due to limited data on multi-lead LV pacing in patients with intermediate QRS duration, the primary endpoint measure in STRIVE HF was feasibility of achieving and maintaining TriV pacing at 6 months and therefore a power calculation was not included in the study protocol. Only patients referred for CRT-D were included in the study as the TriV device with an internal Y-connector was not manufactured in a CRT pacemaker configuration and may have led to selection bias. Quadripolar LV leads were used in the BiV group as standard of care but not in the TriV group given device compatibility limitations which may have introduced bias. Both groups were programmed with AV delays of 100 ms and simultaneous RV–LV pacing as per the study protocol in order to standardize settings for direct comparison (LV1–LV2 delays were not programmable given the parallel Y-configuration). The volumetric response rates in the study for TriV and BiV groups were low and likely represent the underlying substrate with QRS <150 ms and also a high percentage of patients with ICM which is known to result in lower rates of remodelling. Disappointingly, TriV pacing was ineffective in improving CRT response in the ischaemic group, despite the rationale for maximal lead separation in the study protocol was to attempt to create maximal separation between LV electrodes to allow simultaneous recruitment of the largest possible volume of viable LV myocardium compared to a single LV lead. Recruitment was stopped after the 99th patient was randomized as the company no longer manufactured the bespoke TriV pacing device, however, this is unlikely to have affected the results given the target recruitment number was 100 patients. Follow-up data were collected at 6 months and is unknown whether TriV pacing would have resulted in any long-term benefit, however, given the poor response in the TriV group at 6 months and the reduction in battery longevity, this would appear unlikely.

## Conclusion

STRIVE HF was a prospective, multicentre randomized controlled trial specifically designed to assess feasibility and outcome benefits of TriV pacing in patients with LBBB and intermediate QRS duration 120–150 ms. The majority of patients had ICM and response rates were relatively low reflecting the underlying substrate. Implantation of two LV leads was feasible and safe in the short term. Delivery of TriV pacing was feasible at 6-month follow-up in the majority of patients, however, there was no evidence that TriV pacing improved CRT response or provided any clinical benefit to patients with LBBB and intermediate QRS prolongation. Importantly, procedure times were longer and battery longevity was reduced in the TriV group. The current study therefore does not support the practice of multi-lead LV pacing in this patient group.

## Supplementary material

Supplementary material is available at *Europace* online.

### Funding 

MicroPort Cardiac Rhythm Management (formerly LivaNova and Sorin) funded but did not take part in the study design, data collection, analysis, interpretation of results, manuscript writing, or the publication strategy. This work was supported by the Wellcome/Engineering and Physical Sciences Research Council Centre for Medical Engineering (WT203148/Z/16/Z). 

**Conflict of interest:** J.G., B.P., M.K.E., and V.M. have received fellowship funding from Abbott outside of the submitted work. B.S.S. is funded by an National Institute for Health Research project grant and has received speaker fees from EBR systems outside the submitted work. B.J.S. has received a British Heart Foundation project grant outside of the submitted work. S.N. is supported by EPSRC (EP/P01268X/1; NS/A000049/1; EP/M012492/1), BHF (PG/15/91/31812; FS/18/27/33543), NIHR (II-LB-1116-20001), and Wellcome Trust (WT203148/Z/16/Z). Z.W. has received speaker fees for MicroPort and Medtronic and is on the advisory board for Medtronic and Abbott. P.D.L. is supported by UCL/UCLH Biomedicine National Institute for Health Research and Barts Biomedical Research Centre and receives fellowship funding, research grants, and speaker fees from Abbott and Boston Scientific. C.A.R. receives research funding and/or consultation fees from Abbott, Medtronic, Boston Scientific, and MicroPort outside of the submitted work. All other authors declared no conflict of interest.

### Data availability

The authors do not have permission to share the raw data.

## Supplementary Material

euab267_Supplementary_Tables
