# Supplementary material for: Standard care vs. TRIVEntricular pacing in Heart Failure (STRIVE HF): a prospective multicentre randomized controlled trial of triventricular pacing vs. conventional biventricular pacing in patients with heart failure and intermediate QRS left bundle branch block
Source: Europace. 2021 Nov 22;24(5):796–806. doi: 10.1093/europace/euab267 (PMC9071069; doi:10.1093/europace/euab267)
Supplement: euab267_Supplementary_Tables [file euab267_supplementary_tables.docx]

**Supplementary Tables**

**Supplementary Table** **A: Baseline pharmacological therapy**

| **Pharmacological treatment** | **TriV** | **BiV** | **All** | ***p*** |
| --- | --- | --- | --- | --- |
| ACE inhibitor, ARB or Sacubitril with Valsartan | 41(93.2) | 45(95.7) | 86(94.5) | 0.670 |
| Beta-blocker | 44(95.7) | 44(91.7) | 88(93.6) | 0.678 |
| Aldosterone antagonist | 33(86.8) | 30(73.2) | 63(79.7) | 0.166 |
| Loop diuretic | 31(73.8) | 34(77.3) | 65(75.6) | 0.804 |
| Aspirin | 17(53.1) | 22(61.1) | 39(57.4) | 0.625 |
| Clopidogrel | 4(13.8) | 5(15.6) | 9(14.8) | 1.000 |
| Oral anticoagulant | 21(52.5) | 25(62.5) | 46(57.5) | 0.498 |
| Statin | 25(65.8) | 32(74.4) | 57(70.4) | 0.468 |

Values=n(%).

TriV=triventricular; BiV=biventricular; ACE=angiotensin converting enzyme; ARB=angiotensin receptor blocker

**Supplementary Table B: Reverse remodeling outcome measures in the entire cohort and subgroups of atrial fibrillation and heart failure etiology**

| **Number of volumetric responders for the entire cohort and prespecified subgroups:** | **TriV** | **BiV** | **All** | ***p*** |
| --- | --- | --- | --- | --- |
| Entire cohort | 15(32.6) | 21(42.9) | 36(37.9) | 0.398 |
| Permanent Atrial fibrillation | 3(27.3) | 6(50.0) | 9(39.1) | 0.400 |
| Sinus rhythm | 12(35.3) | 14(40.0) | 26(37.7) | 0.805 |
| Ischaemic cardiomyopathy | 5(20.0) | 10(33.3) | 15(27.3) | 0.366 |
| Non-ischaemic cardiomyopathy | 10(47.6) | 11(57.9) | 21(52.5) | 0.545 |

Values=n(%). Volumetric response was defined as ≥15% reduction in LVESV on two-dimensional transthoracic echocardiography.

TriV=triventricular;BiV=biventricular;LVESV=left ventricular end-systolic volume

**Supplementary Table C: Left ventricular end-systolic volumes at baseline and six-month follow-up for heart rhythm and heart failure etiology subgroups**

| **Variable** | **TriV** | **BiV** | ***p*** |
| --- | --- | --- | --- |
| **LV end-systolic volume(mL) for SR subgroup** | | | |
| Baseline | 125.8±66.1 | 131.0±49.7 |  |
| Follow-up | 117.2±74.8 | 110.0±56.5 |  |
| Absolute change(mL) | -8.6±29.2 | -21.0±56.3 | 0.296 |
| Percentage change(%) | -9.0±26.9 | -12.2±39.6 | 0.736 |
| **LV end-systolic volume(mL) for Permanent AF subgroup** |  |  |  |
| Baseline | 170.7±77.2 | 142.9±61.1 |  |
| Follow-up | 124.6±49.9 | 97.9±45.0 |  |
| Absolute change(mL) | -46.1±61.3 | -45.0±58.0 | 0.972 |
| Percentage change(%) | -23.6±21.0 | -28.1±32.5 | 0.755 |
| **LV end-systolic volume(mL) for ICM subgroup** | | | |
| Baseline | 138.7±72.6 | 122.2±30.3 |  |
| Follow-up | 135.2±75.4 | 109.9±53.1 |  |
| Absolute change(mL) | -3.5±27.7 | -12.3±56.9 | 0.501 |
| Percentage change(%) | -2.7±23.6 | -6.9±43.1 | 0.680 |
| **LV end-systolic volume(mL) for NICM subgroup** | | | |
| Baseline | 126.3±62.7 | 145.2±71.3 |  |
| Follow-up | 88.0±43.3 | 102.1±54.4 |  |
| Absolute change(mL) | -38.4±47.0 | -43.0±52.4 | 0.738 |
| Percentage change(%) | -28.6±21.8 | -26.2±28.2 | 0.806 |

Values=mean±SD. Absolute and percentage change values are the difference between values obtained from baseline and 6-month follow-up measures.

TriV=triventricular;BiV=biventricular;AF=atrial fibrillation;SR=sinus rhythm;ICM=ischaemic cardiomyopathy;NICM=non-ischaemic cardiomyopathy;LV=left ventricular
